# The pericardium horror show

**DOI:** 10.1186/2036-7902-4-S1-A22

**Published:** 2012-12-18

**Authors:** Ramon Nogué Bou, J Fabregat, R Vilella, A Encinas, T Villen, R Campos

**Affiliations:** 1 Hospital Montserrat, University of Lleida, Alcalde Sol, Nº 6-3. Lleida, Spain

## 

We describe three cases where the ultrasonography "point of care" was decisive.

### Case 1

83 year old woman with a history of hypertension and stroke, who were admitted to our hospital for right hemiplegia with an evolution of more than 3 hours. She shows us a report that two days earlier was admitted in another center for an episode of atrial fibrillation at 150 bpm of less than 48 hours of evolution, where he underwent electrical cardioversion reverting to sinus rhythm at 72 bpm and anticoagulation with heparin of low molecular weight was started.

Physical examination: BP 114/76 mmHg, HR 72 bpm, Temp: 36.6 ° C, Sat 02: 97%.

Cardiac auscultation: arrhythmic tones, systolic murmur. Jugular venous distention. Right hemihipoestesia and hemiparesia, dysarthria. No other remarkable findings.

Evolution: Diagnosed with stroke, is admitted to our center. During the following day she presented several episodes of hypotension which responded to fluid therapy. An ultrasonography guided central line placement, for better control was decided. At this point significant jugular distension and slow and turbulent flow visible without Doppler was detected.

Given these findings without clear signs of cardiac failure, an obstruction at lower level was suspected. We decided to perform an echocardiography. A pericardial effusion, which was not present in an echocardiography performed ten days ago, was detected.

Clinical trial: Pericardial effusion post-cardioversion.

Treatment: We performed ultrasound-guided pericardiocentesis, obtaining 160ml of sero-hematic liquid. After that the patient showed progressive improvement with recovery of blood pressure levels.

### Case 2

82 year old woman with a history of 3rd degree AV block with a pacemaker placed 20 days before the current admission.

She complains of dizziness similar to those presented before pacemaker implantation, accompanied by thoracic discomfort.

Physical examination: TA: 100/80 mmHg, HR 80 bpm, O2 Sat: 94%.

Cardiac auscultation: rhythmic sounds, without murmurs. Lung auscultation: basal crackles. Jugular venous distention. No other remarkable findings.

Investigations: ECG: 3rd degree AV block, left bundle branch block with HR 78 bpm. Peacemaker spikes not correlated with P waves or QRS complexes.

Chest radiography: cardiomegaly. Peacemaker catheter on right ventricle.

Evolution: Given the findings on ECG, we decided to perform an echocardiogram to check the placement of the pacemaker, although it appears correct in the chest radiograph. We found the end of the catheter into the pericardium,, accompanied with a minimal effusions an a perforation of the right ventricle wall.

Treatment: We call the cardiologist, who retired the catheter.

### Case 3

43 year old woman presented hemodynamic deterioration during the postoperative of hemicolectomy for colon cancer.

Physical examination: TA: 89/58 mmHg, HR 134 bpm.

Abdomen soft, depressible. No pathological products present into colostomy bag. Jugular venous distention. No other remarkable findings.

Evolution: Assuming diagnosis of hypovolemic shock fluid therapy was initiated, with worsening of the hemodynamic status. The surgeon did not found any abdominal or surgical complication.

Given the presence of jugular venous distention, echocardiography was decided, objectifying pericardial effusion. In ultrasound guided pericardiocentesis a transparent liquid was extracted. In biochemistry showed an ion concentration similar to physiologic saline.

Diagnostic judgment: Central line catheter complication.

Treatment: Given the findings fluid infusion was suspended, and central line retired 3 cm, presenting hemodynamic improvement. It is therefore assumed that the central line was placed in the pericardium, being the cause of the effusion.

## Conclusions

The three cases have exposed three facts in common: first, the presence of pericardial effusion initially unsuspected. Second, the difficulty to suspect this condition without ultrasonography. And third, its easy detection by bedside echocardiography, which changed the treatment, avoiding potential aggravation.

This highlights the important role of ultrasonography and its progressive introduction in the emergency services, enabling rapid detection of diseases by the staff using protocols.

